# Induction of mitochondria-mediated apoptosis and PI3K/Akt/ mTOR-mediated autophagy by aflatoxin B_2_ in hepatocytes of broilers

**DOI:** 10.18632/oncotarget.13356

**Published:** 2016-11-15

**Authors:** Binlong Chen, Diyan Li, Miao Li, Sichen Li, Kenan Peng, Xian Shi, Lanyun Zhou, Pu Zhang, Zhongxian Xu, Huadong Yin, Yan Wang, Xiaoling Zhao, Qing Zhu

**Affiliations:** ^1^ Farm Animal Genetic Resources Exploration and Innovation Key Laboratory of Sichuan Province, Sichuan Agricultural University, Chengdu, P.R.China, 611130

**Keywords:** aflatoxin B_2_, mitochondria-mediated apoptosis, PI3K/Akt/mTOR-mediated autophagy, hepatocytes, broiler

## Abstract

Aflatoxins have been shown to induce hepatotoxicity in animal models, but the effects of aflatoxin B_2_ (AFB_2_) on broiler hepatocytes is unclear. This study aimed to investigate the effects of AFB_2_ on apoptosis and autophagy to provide an experimental basis for understanding the mechanism of aflatoxin-induced hepatotoxicity. One hundred-twenty Cobb500 broilers were allocated to four groups and exposed to 0 mg/kg, 0.2 mg/kg, 0.4 mg/kg, and 0.8 mg/kg of AFB2 per day for 21 d. AFB_2_ exerted potent proapoptotic and proautophagic effects on hepatocytes, with increased numbers of apoptotic and autophagic hepatocytes.

Poly ADP-ribose polymerase (PARP) was cleaved and caspase-3 was activated in experimental groups, showing that the apoptosis of hepatocytes was triggered by AFB_2_. Increased levels of the autophagy factors Beclin-1 and LC3-II/LC3-I, as well as down-regulation of p62, a marker of autophagic flux, provided additional evidence for AFB_2_-triggered autophagy. AFB2 induced mitochondria-mediated apoptosis via the production of reactive oxygen species (ROS) and promotion of the translocation of Bax and cytochrome c (cyt c) between mitochondria and the cytosol, triggering the formation of apoptosomes. AFB2 also inhibited the phosphoinositide 3-kinase/Akt/mammalian target of rapamycin (PI3K/Akt/mTOR) pathway by activating PI3K, Akt, and mTOR and inhibiting their phosphorylation, contributing to the proautophagic activity of AFB_2_. These findings provide new insights into the mechanisms involved in AFB_2_-induced hepatotoxicity in broilers.

## INTRODUCTION

Aflatoxins are known to have strong hepatotoxic and carcinogenic effects and contaminate a wide variety of tropical and subtropical foodstuffs [1]. Aflatoxins are difuranocoumarin compounds, which include B_1_, B_2_, G_1_, G_2_, M_1_, and M_2_. The toxicity of aflatoxin B_1_ (AFB_1_) has been shown to be higher that that of other common aflatoxins [2, 3]. Although aflatoxins are deleterious to poultry, aflatoxin-contaminated feed is practically unavoidable. Many reports have demonstrated the immunosuppressive action of AFB_1_ and resulting humoral and cellular responses [4, 5]. Previous studies indicated that low doses of AFB_1_ produced such responses in poultry and that poultry were extremely sensitive to the toxic effects of AFB_1_. Research also showed that the consumption of AFB_1_-contaminated feed by poultry had various adverse effects, including liver damage, immunosuppression, and poor growth [6]. In addition, the administration of AFB_1_ to developing chickens caused DNA damage in the liver, morphological defects, and embryonic mortality [7]. Recent reports revealed that AFB_1_ led to increased levels of apoptotic splenocytes and pathological changes, resulting in spleen damage. Such as Wolzak *et al*. reported that residues of aflatoxins were highest in kidney and liver tissue when broilers were exposed to a mixture of AFB_1_ and AFB_2_ for 4 wk [8]. Peng xi *et al*. found that broilers given corn contaminated with AFB_1_ and AFB_2_ induced pathological changes in the spleen, splenocyte apoptosis, cell cycle blockage, and up-regulation of CD8^+^ T cells [9].

Although hepatotoxicity in broilers and ducklings exposed to AFB_1_ has been reported, only few studies have explored the relationship among autophagy, apoptosis, and AFB_2_ in the liver. The present study aimed to investigate the effects of AFB_2_ on apoptosis and autophagy in livers of broilers to provide experimental evidence of the potential molecular mechanisms underlying AFB_2_-induced hepatotoxicity.

## RESULTS

### AFB_2_ triggered apoptosis of hepatocytes

**Figure 1:**
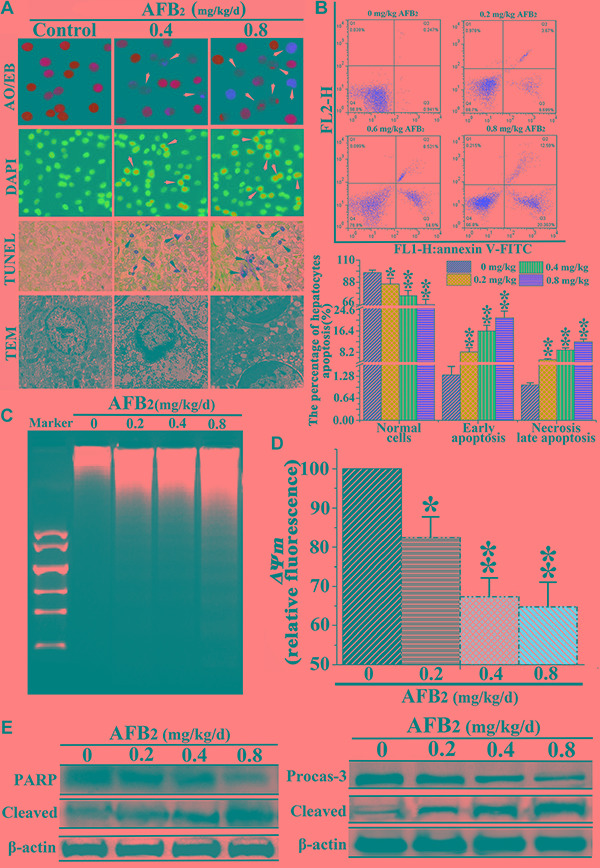
Effect of AFB_2_ on apoptosis of hepatocytes (**A**) Showing normal and early apoptotic cells stained by AO (green fluorescence) and late apoptotic cells stained by EB (red fluorescence) (200×). Nuclear morphological changes in hepatocytes were observed using a fluorescence microscope after DAPI (highlight, arrow). TUNEL-positive hepatocytes are shown (black arrow, 200×). Ultrastructural observations of swainsonine-treated cells visualized under a transmission electron microscope (black arrow, 11000×). **(B)** A scattergram of apoptotic hepatocytes analyzed using flow cytometry after annexin V and PI staining. **(C)** Induction of DNA fragmentation. The DNA fragmentation of broilers’ hepatocytes were measured via 2% agarose gel electrophoresis, followed by visualization of bands and photography. **(D)** AFB_2_ induced the collapse of *ΔΨm*. The cell suspension was filtered through 300-mesh nylon and then stained with JC-1, followed by FCM analysis. **(E)** The protein levels of PARP and caspase-3 examined by a Western blot analysis. The data are presented as the means ± SD of three independent experiments. *p < 0.05 and **p < 0.01 compared with the control group.

Apoptosis, an essential physiological cell death process that occurs during various physiological and pathological conditions [10, 11], can be promoted by toxic stimuli [12]. To examine AFB_2_-induced apoptosis of hepatocytes in broilers, morphological changes in hepatocytes subject to apoptosis were determined using a TUNEL assay, DAPI staining, and TEM. In addition, apoptosis-associated factors PARP and caspase-3 were detected via a Western blot. As shown by DAPI and AO/EB staining, the hepatocytes in the experimental groups showed typical apoptotic nuclei, including nuclear fragmentation, cell membrane destruction, and chromatin condensation (white arrow, Figure 1A). The TUNEL assay, which was performed to detect DNA strand breaks that occurred prior to nuclear fragmentation, revealed significant apoptosis in the experimental groups as compared to the control group. TEM further confirmed the apoptotic features. High-power TEM views indicated that chromatin condensation occurred along the nuclear membrane, producing a crescent pattern in early apoptosis. Thereafter, the chromatin condensed into solid, rounded masses, which underwent fragmentation, whereas the nuclei of the controls were normal (black arrow, Figure 1A). The FEM analysis of the apoptotic hepatocytes indicated that the percentage of apoptotic hepatocytes increased significantly, whereas that of normal hepatocytes decreased markedly in a dose-dependent manner, indicating that AFB2 inhibited hepatocyte growth (Figure 1B). The analysis of DNA fragmentation indicated that the DNA ladder appeared to be more evident with increasing doses of AFB2, whereas no DNA fragments were observed in the control (Figure 1C). The collapse of the *ΔΨm* was also observed using FCM (Figure 1D). The Western blot analysis showed that PARP, an indicator of the activation of caspase-3, which is a key executioner caspase in the apoptosis pathway, was obviously cleaved [13, 14]. The Western blot showed that full-length procaspase-3 decreased in a dose-dependent manner, whereas the cleaved form increased, demonstrating the induction of apoptosis (Figure 1E). The results suggested that AFB_2_ inhibited hepatocyte growth in broilers by inducing the apoptosis of hepatocytes in a dose-dependent manner.

### Autophagy of hepatocytes was triggered by AFB_2_

**Figure 2:**
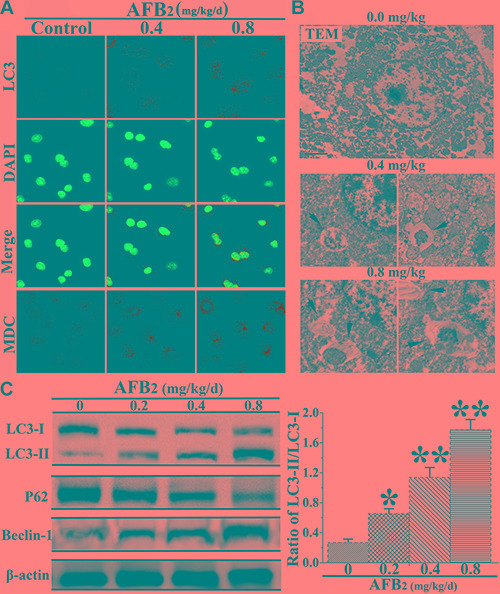
Effect of AFB_2_ on autophagy of hepatocytes in broilers (A) Hepatocytes stained with MDC (bright color) and LC3 (green) antibody using a fluorescence microscope (200×), respectively. Nuclei were stained with DAPI (blue) (bar: 10 μm). **(B)** Morphological observation of autophagy in hepatocytes, showing the characteristic ultrastructural morphology of autophagy, such as autophagic vacuoles (black arrow, ×12000, in hepatocytes. **(C)** Representative blots showing the expression levels of LC3-I, LC3-II, p62, and Beclin-1 in hepatocytes. β-actin was used as an internal control. The bar graph shows the ratio of LC3-II/LC3-I. The data are presented as the means ± SD of three independent experiments. *p < 0.05 and **p < 0.01 compared with the control group.

After the observation of AFB_2_-induced apoptosis in hepatocytes, the effect of AFB_2_ on autophagosome formation in hepatocytes was examined using confocal microscopy, TEM, and Western blot analyses. Furthermore, MDC and DAPI staining were performed, in addition to LC3 immunostaining using fluorescent antibodies to LC3, to confirm autophagy induced by AFB_2_. The increased subcellular localization of punctate LC3 was detected in the hepatocytes of the experimental groups. The formation of LC3 puncta increased in a dose-dependent manner. Increased fluorescence intensity of the MDC-stained cells in the AFB_2_-administered groups pointed to more extensive MDC-positive autophagic vacuoles in the experimental groups compared to the control group (Figure 2A). In the experimental groups, the results of TEM revealed cells with an ultrastructural morphology typical of autophagy, including abundant autophagic vacuoles sequestered in the cytoplasm and organelles, such as mitochondria and endoplasmic reticulum (Figure 2B). To further ascertain the formation of autophagosomes in hepatocytes, a Western blot analysis of three major autophagy factors, LC3, Beclin-1, and P62, was performed. Autophagy is tightly regulated by Beclin-1, and it serves as a platform for the recruitment of ATGs, which are critical for autophagosome formation [15, 16]. The results showed that the level of Beclin-1 was markedly elevated in in hepatocytes. The expression of LC3-II increased in a concentration-dependent manner, whereas that of LC3-I decreased, resulting in an increased ratio of LC3-II/I. As shown in Figure 2C, the level of the p62 protein, a marker of autophagic flux [17], was markedly decreased by the AFB_2_ treatment in a dose-dependent manner. The results indicated that AFB_2_ induced autophagosome formation in hepatocytes.

### The mitochondrial apoptotic pathway was activated by AFB_2_

**Figure 3:**
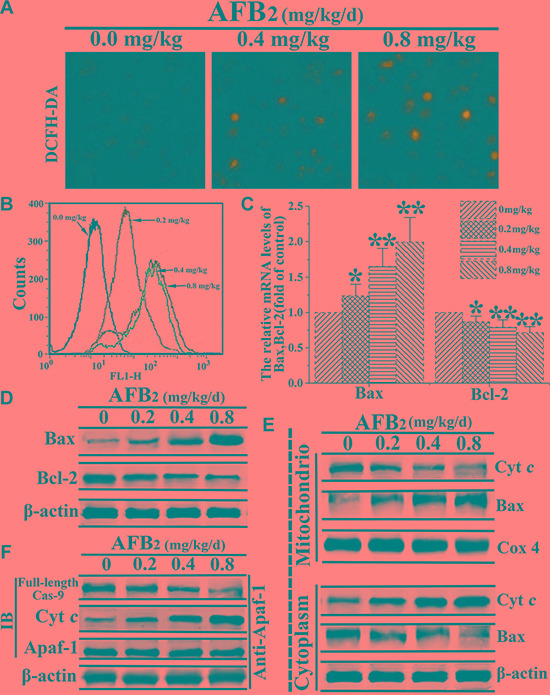
AFB_2_-induced apoptosis of hepatocytes via activation of the mitochondria-dependent pathway (**A**) DCFH-DA staining and the detection of intracellular ROS levels in hepatocytes of broilers given AFB_2_. **(B)** Intracellular ROS levels determined using H2DCFDA staining. The histogram of FCM showing the dose-dependent increase in the probe fluorescence intensity. **(C–D)** Detection of mRNA levels and protein levels of Bax and Bcl-2 following the extraction of total RNA and protein from hepatocytes via a quantitative real-time polymerase chain reaction (qRT-PCR) assay and Western blot analysis, respectively. **(E)** Results of the Western blot analyses of the proteins extracts from the mitochondrial and cytosolic fractions of hepatocytes used to measure the translocation of Bax and cytochrome *c* (cyt *c*). COX 4 and β-actin were used as internal controls for the mitochondrial and cytosolic fractions, respectively. **(F)** AFB_2_ induced the formation of apoptosomes. Protein extracts from hepatocytes were collected and the immunoprecipitation assays against Apaf-1 were performed to detecte the protein levels of full-length caspase-9 and cyt *c* via western blot aiming to indicate the formation of the apoptosome complex. All the data are presented with the means ± SD and mean values of three independent experiments. *p < 0.05, compared with the control group; **p < 0.01, compared with the control group.

Mitochondria are known to play an important role in the intrinsic pathway of mammalian apoptosis by releasing death factors, such as cytochrome c (cyt *c*), into the cytosol [18]. The detachment of cyt *c* from cardiolipin is triggered by an increase in intracellular ROS, which is one of the early events of apoptosis. To detect mitochondria-mediated apoptotic factors, changes in ROS levels following AFB_2_ administration at different doses were analyzed by DCFDA staining and FCM analysis. The FCM analysis of DCFDA fluorescence revealed low intracellular ROS levels in the 0 mg/kg AFB_2_ group but profound increases in the 0.4 mg/kg and 0.8 mg/kg AFB_2_ groups (Figure 3A and Figure 3B). The expression of both protein and RNA levels of Bax was up-regulated, whereas that of Bcl-2 was down-regulated according to the dose administered. This result suggested that AFB_2_ might induce an increase in the Bax/Bcl-2 ratio due to the collapse of the ΔΨm and release of mitochondrial proapoptotic factors (Figure 3C and 3D). To further explore whether the apoptosis of hepatocytes was associated with the translocation of Bax and release of mitochondrial proapoptotic molecules, the protein extracts from both the mitochondrial and cytosolic fractions of the hepatocytes were subjected to Western blot analyses. The results indicated that Bax levels increased in the mitochondrial fraction, whereas cyt *c* levels decreased, concomitant with decreased Bax and increased cyt *c* levels in the cytosolic fraction (Figure 3E). Next, to detect whether AFB_2_ promoted the formation of apoptosomes, the cell lysates were immunoprecipitated with an anti-Apaf-1 antibody and subsequently subjected to a Western blot analysis with anti-caspase-9 and anti-cyt *c* antibodies. As shown in Figure 3F, Apaf-1 interacted with caspase-9 and cyt *c*. These results suggested that AFB_2_-induced apoptosis occurred mainly via the activation of the mitochondrial pathway.

### AFB_2_ induced autophagy in hepatocytes by inhibiting the PI3K/Akt/mTOR pathway

**Figure 4:**
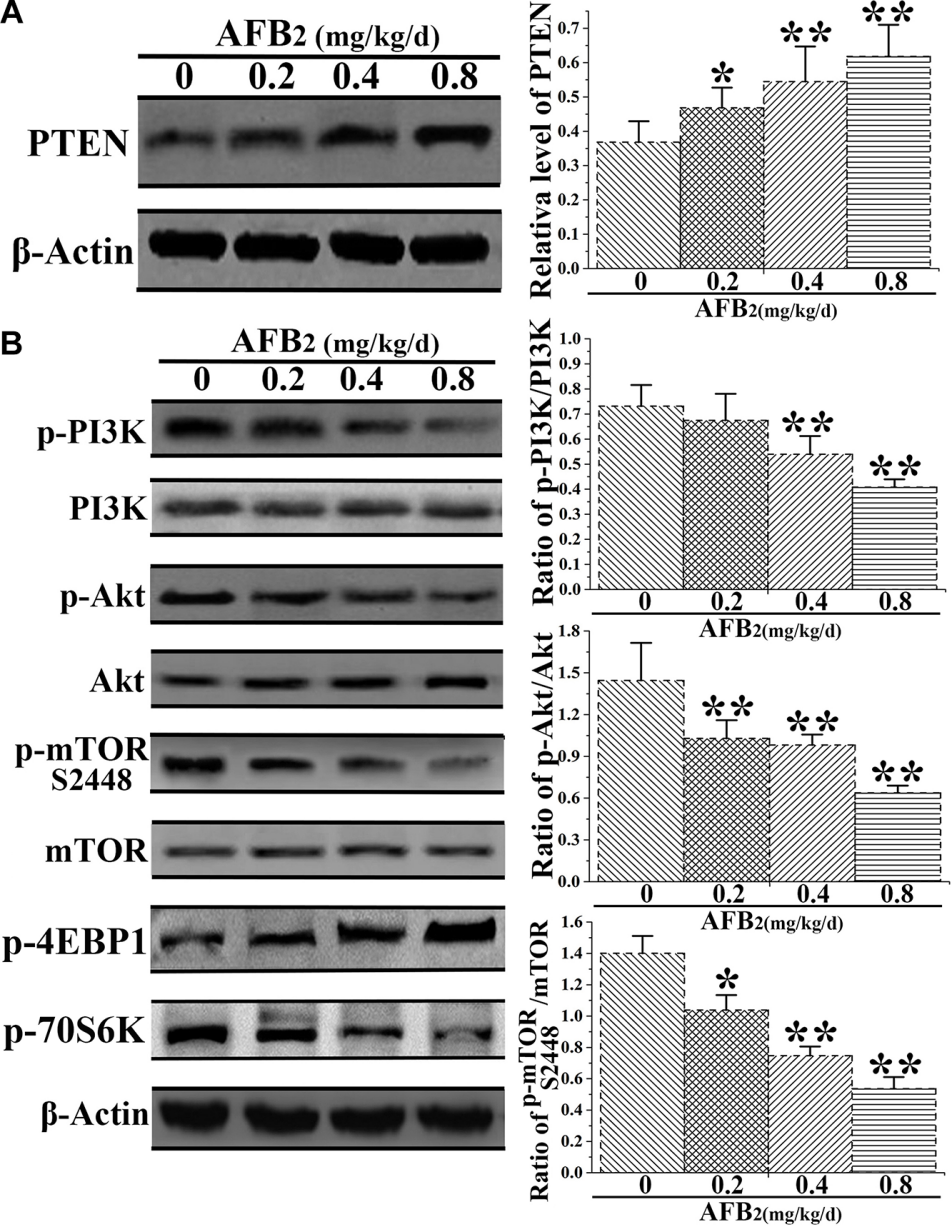
Effect of AFB_2_ on phosphoinositide 3-kinase/Akt/mammalian target of rapamycin (PI3K/Akt/mTOR)-mediated autophagy in hepatocytes (**A**) Protein extracts from hepatocytes were collected, and the protein level of PTEN was analyzed via a Western blot. The bar graph shows the relative level of PTEN. **(B)** Representative blots showing the expression levels of p-PI3K, PI3K, p-Akt, Akt, p-mTOR S2448, and mTOR in hepatocytes of broilers administered AFB_2_. The bar graphs show the ratio of p-PI3K/PI3K, p-Akt/Akt, p-mTOR S2448/mTOR, p-4EBP1, and p-70S6K in the hepatocytes. All the data are presented with the means ± SD and mean values of three independent experiments. *p < 0.05, compared with the control group; **p < 0.01, compared with the control group.

Autophagy is an intracellular process, which delivers cytoplasmic components to autophagosomes and lysosomes for degradation [19, 20]. A previous study implicated the phosphoinositide 3-kinase/Akt/mammalian target of rapamycin (PI3K/Akt/mTOR) signaling pathway and ROS in the regulation of autophagy [21]. Thus, the present study investigated the mechanisms underlying the autophagy-inducing effect of AFB_2_ in hepatocytes of broilers. Previous research demonstrated that phosphatase and the tensin homolog (PTEN), a tumor suppressor gene, downregulated PI3K activity by converting PIP3 back to PIP2, thereby controlling the induction of autophagy [22]. The results indicated that AFB_2_ administration increased the expression level of PTEN in hepatocytes of broilers in a concentration-dependent manner (Figure 4A). Although it did not significantly affect the protein expression of total PI3K, it reduced the expression of phosphorylated-PI3K (p-P13K), resulting in a concentration-dependent decrease in the level of p-PI3K relative to that of total PI3K in the hepatocytes. To further dissect this pathway, the expression levels of major downstream autophagy-related proteins Akt and mTOR were examined. Recent studies demonstrated that mTORC1 contained primarily Ser2448 phosphorylation. In common with the decrease in the level of p-PI3K, AFB_2_ also resulted in substantial downregulation of phosphorylated Akt (p-Akt) and phosphorylated mTOR S2448 (p-mTORC1), but without affecting the protein levels of Akt and mTOR, resulting in a decreased p-Akt/Ak:p-mTOR S2448/mTOR ratio (Figure 4B). Together, the results suggested that AFB_2_-induced autophagy of hepatocytes in broilers occurred via a PI3K/Akt/mTOR-mediated autophagy pathway.

## DISCUSSION

Aflatoxins, secondary fungal metabolites, are largely produced by the fungi *Aspergillus flavus* and *Aspergillus parasiticus* [23]. Many aflatoxins and their metabolites have adverse side effects. AFB_2_ is a toxic mycotoxin, which is thought to have harmful hepatotoxic, mutagenic, carcinogenic, and teratogenic effects on many species of livestock [9]. Although studies have examined the apoptosis of hepatocytes in broilers induced by AFB, only a few reports have focused on the relationship between autophagy and AFB_2_ in the liver and the functional role of autophagy in AFB_2_-induced hepatotoxicity. Therefore, the present study was conducted to explore the molecular mechanism of apoptosis and AFB_2_-induced autophagy.

The present study showed for the first time that AFB_2_-induced hepatotoxicity in broilers was characterized by the induction of apoptosis and autophagy of hepatocytes via mitochondria- and PI3K/Akt/mTOR-mediated pathways, respectively. The observations of morphological characteristics, including cellular shrinkage, chromatin condensation, DNA fragmentation, phosphatidylserine externalization, and autophagosomes, confirmed that AFB_2_ induced apoptosis and autophagy of hepatocytes in broilers. The activation of caspase-3, a key executioner caspase in the apoptosis pathway [14, 24], and PARP, an indicator of caspase-3 activation during apoptosis, indicated that the apoptosis of hepatocytes was induced by AFB_2_. MDC and LC3 are specific markers of autophagic vacuoles and autophagosomes, respectively, and the formation of LC3 puncta in autophagosomes can be observed by confocal microscopy [25]. In the present study, extensive MDC-positive autophagic vacuoles and increased subcellular localization of LC3 punctate clearly showed that AFB_2_ induced autophagosome formation in hepatocytes. Beclin-1 functions in autophagy and is necessary for the formation of autophagosomes during the autophagic sequestration process. LC3-II plays indispensable roles in autophagosome formation and is the most reliable biomarker of autophagy [26]. The decreased protein level of p62, up-regulation of the expression level of the Beclin-1 protein, and increase in the ratio of LC3-II/LC3-I demonstrated that AFB_2_ triggered autophagy in hepatocytes. These results suggested that AFB_2_ may serve as an inducer of hepatocyte apoptosis and autophagy, leading to liver impairment in broilers.

The central role of the mitochondrial pathway in the intrinsic apoptosis pathway, which determines whether cells survive or die, is well known [27, 28]. Bcl-2 and Bax are mainly responsible for regulating the integrity of the mitochondrial membrane[29]. In the present study, the following findings suggested that AFB_2_ triggered dysregulation of the mitochondrial apoptotic pathway, mediated by Bcl-2 and Bax: 1) the formation of apoptosomes containing Apaf-1, cyt *c*, and caspase-9 and 2) the release of cyt *c* from dysfunctional mitochondria into the cytosol and subsequent formation of apoptosomes with the apoptotic proteases Apaf-1 and caspase-9. The dysregulation of mitochondrial function typically results in increased generation of ROS [30]. The increase in intracellular ROS in the present study suggested that the mitochondria-mediated apoptosis pathway was activated.

The PI3K/Akt/mTOR pathway is a key signaling pathway, which is induced by various receptor-tyrosine kinases and acts as a central pathway in the promotion of cell growth, motility, survival, and metabolism [31]. PI3K activates Akt, which, in turn, results in the phosphorylation and activation of mTOR. PTEN can inhibit Akt/mTOR signaling [32]. In the current study, the data showed that AFB_2_ triggered PTEN and inhibited the phosphorylation of PI3K, Akt, and mTOR, leading to the induction of PI3K/Akt/mTOR-mediated autophagy in hepatocytes of broilers. Together, these findings provided evidence that AFB_2_ promoted autophagy via the activation of PTEN and AMPK and the inhibition of the PI3K/Akt/mTOR signaling pathway, thereby contributing to the autophagy-inducing activities of AFB_2_. According to a previous study [33], the inhibition of the PI3K/Akt/mTOR pathway seemed to protect hepatocytes from AFB_2_ and promote hepatocyte survival.

Bcl-2 and Beclin-1 are involved in the regulation of both autophagy and apoptosis. In response to specific signals, Beclin-1 also plays a vital role in the convergence between autophagy and apoptosis. Bcl-2 inhibits Beclin-1-dependent autophagy via its interaction with Beclin-1 [34]. So the results that Beclin 1 was activated and Bcl-2 was suppressed by AFB_2_ suspected that AFB_2_ regulated both Beclin-1 and Bcl-2 to trigger the autophagy and apoptosis of hepatocytes. Genetic manipulation of the autophagy-mediated pathway indicated that autophagy might be a protagonist of apoptosis [35]. In the present study, AFB_2_ triggered both autophagy and apoptosis of hepatocytes in broilers. Thus, we speculate that overactivation of autophagy during unchecked degradative processes might contribute to apoptosis in hepatocytes. Previous reports showed that cellular function collapsed when the activation of autophagy exceeded a certain threshold, potentially contributing to apoptosis. Considering the aforementioned finding, it is possible that the autophagy triggered by AFB_2_ caused cytotoxicity of hepatocytes and that autophagy preceded apoptosis.

In summary, the present study demonstrated that AFB_2_ markedly induced apoptosis via the activation of the mitochondrial pathway by triggering ROS and down-regulating Bcl-2, thereby promoting the translocation of Bax into mitochondria. This resulted in the release of cyt *c* into the cytosol, activation of caspases-9 and -3, and cleavage of PARP. AFB_2_ triggered autophagy by activating PTEN and suppressing p-PI3K, p-Akt, and p-mTOR, pointing to inhibition of the PI3K/Akt/mTOR pathway. This study provides new insight into the mechanisms of mitochondria-mediated apoptosis and PI3K/Akt/mTOR-mediated autophagy caused by AFB_2_.

## MATERIALS AND METHODS

### Ethics statement, animals, and diets

All the experimental procedures in the present study complied with the recommendations of the Animal Care and Use Committee of Sichuan Agricultural University, and the study was approved by the Animal Care and Use Committee, Sichuan Agricultural University, Sichuan, China (permit no.: DKY-B20100805.

The study consisted of one hundred-twenty 1-d-old healthy broilers (obtained from Wenjiang Poultry Farm, Sichuan Province, China). The broilers were randomly divided into four equal sized groups and given 0 mg/kg/d (control), 0.2 mg/kg/d, 0.4 mg/kg/d, and 0.8 mg/kg/d AFB_2_ for 21 d. They were housed in cages with electrically heated units and provided with water. Nutritional requirements were adequate according to the 1994 National Research Council guidelines and Chinese chicken feeding standards (NY/T33-2004).

### DNA fragmentation assay

Hepatocytes from the control and AFB_2_-administered groups were washed with phosphate-buffered saline (PBS). DNA extraction was performed as described in a previous study [36]. After dissolving the DNA in Tris-EDTA(TE) buffer, it was subjected to 2% agarose gel electrophoresis for DNA fragmentation analysis.

### Annexin-V/propidium iodide (Annexin-V/PI) apoptosis detection by flow cytometry

The cell suspension was filtered through a 300-mesh nylon screen and washed twice with cold PBS. The cells were then suspended in a 1× binding buffer (Cat. No. 51-66121E) at a concentration of 1 × 10^6^ cells/mL. Then, 100 μL of the solution were transferred to a 5-mL culture tube, and 5 μL of annexin V-FITC (Cat. No. 51-65874X) and 5 μL of PI (Cat. No. 51-66211E) were added. The cells were then gently vortexed and incubated for 15 min at room temperature (25°C) in the dark. Finally, 400 μL of 1×binding buffer were added to each tube and analyzed by flow cytometry (BD FACSCalibur) within 1 h.

### Quantitative real-time polymerase chain reaction (qRT-PCR) analysis

Total RNA was isolated from the liver powder (50 mg) using Trizol (Aidlab, China). The synthesis of single-stranded cDNA from 5 μg of RNA was performed using a TUREscript 1st strand cDNA Synthesis Kit (Aidlab, China), and mRNA was reverse transcribed into cDNA. The cDNA was used as a template for qRT-PCR analysis. Relative gene expression was defined as the ratio of the target gene expression versus β-actin gene expression [37]. The primer sequences were as follows: ACCATGCCCGTGCGTTTTG (forward) and ATGATGGCGTAGACCTTGCGGATAA (reverse) for Bax; AGCAGCTAAGCCCCACAAAAACA (forward) and AGGGCGCTCAGTGCAGGTATCAG (reverse) for Bcl-2. The gene expression fold changes were calculated using cycle time values [38].

### Western blot analysis

The hepatocytes were harvested, washed with ice-cold PBS, and then lysed with ice-cold RIPA lysis buffer (Beyotime Inst. Biotech) with 1 mmol/L of PMSF. Protein concentrations were calculated using BCA assay kits (Pierce). Total cellular protein (20 μg) was subjected to 12% SDS-PAGE and transferred to PVDF membranes (Millipore). The membranes were blocked with 5% defatted milk powder at room temperature for 1 h, followed by immunoblotting with primary antibodies at 4°C overnight and immunoblotting with HRP-conjugated secondary antibody at room temperature for 1 h. Following each step, the membranes were washed five times with PBS-T for 3 min. Finally, the blots were developed using an enhanced chemiluminescence system (Pierce).

### 4′,6-diamidino-2-phenylindole (DAPI) and Acridine orange/Ethidium bromide (AO/EB) staining

The livers were minced using scissors to form a cell suspension, which was filtered through a 300-mesh nylon screen. For DAPI staining, the hepatocytes were fixed with 80% ethanol at room temperature for 30 min. The fixative was removed, and the hepatocytes were washed with PBS three times. They were then incubated with DAPI (1 μg/ml) for 45 min at room temperature in the dark. For AO/EB staining, cells without fixative were loaded with a 100 μl freshly prepared AO/EB staining solution (100 μg/ml) and observed under a Nikon fluorescence microscope (Nikon Inc.) in less than 20 min.

### Monodansylcadaverine (MDC) staining of autophagic vacuoles

The livers were minced using scissors to form a cell suspension, which was filtered through a 300-mesh nylon screen. Autophagic vacuoles were labeled with 0.05 mmol/L of MDC in PBS at 37°C for 10 min. The cells were then washed three times with PBS. Autophagic vacuoles in the hepatocytes were observed under a fluorescence microscope (Olympus, BX-60). The fluorescence intensity of MDC was measured at an excitation wavelength of 380 nm and emission wavelength of 530 nm.

### Immunocytochemistry

The hepatocytes were fixed with 3% paraformaldehyde for 15 min at 37°C. Next, a permeabilization step was carried out using chilled methanol (100%) for 10 min at −20°C. The cells were then incubated in a blocking solution containing 5% bovine serum albumin and 1% Triton-X 100 for 1 h at 37°C. The cells were incubated with LC3 antibody for 12 h at 4°C, followed by incubation with FITC-conjugated secondary antibody for 1 h at 37°C. The nuclei were stained with DAPI (1 μg/ml) for 10 min at 37°C. Fluorescence images were captured using an LSM 700 confocal laser scanning microscope (Carl Zeiss).

### TdT-mediated dUTP nick end labeling (TUNEL) assay

Hepatic tissue was fixed in 4% paraformaldehyde, embedded in paraffin, and cut into 6 μm sections. To detect DNA fragmentation, a TUNEL assay was performed using an in situ cell death detection kit (Vazyme) according to the manufacturer's instructions. After mounting TUNEL-positive cells, the nuclei were counterstained with DAPI, and the sections were observed at ×400 magnification under a Nikon microscope (Nikon Inc.).

### Transmission electron microscopy (TEM) observations

Ultrastructural morphology changes were observed under a transmission electron microscope. After AFB_2_ administration, the cells were fixed with 3% glutaraldehyde and postfixed with 1% OsO4. The samples were then dehydrated in graded ethanol solutions, followed by embedment and sectioning. Ultrathin sections were stained with uranyl acetate and lead citrate and then observed under a transmission electron microscope (JEM-1230) at 60 kV.

### Mitochondrial transmembrane potential (*ΔΨm*) assessment

The transmembrane potential *ΔΨm* was analyzed using a JC-1 Mitochondrial Potential Detection Kit (Biotium Inc.) by FCM. The cell suspension was filtered through a 300-mesh nylon mesh, washed twice with cold PBS, and stained by 5,5′,6,6′-tetrachloro-1,1′,3,3′ tetraethylbenzimidazolcarbocyanine iodide (JC-1; Molecular Probes) in PBS for 15 min at room temperature in the dark, followed by flow cytometric analysis.

### Measurement of intracellular reactive oxygen species (ROS)

Intracellular ROS levels were measured using 5,6-chloromethyl-2′,7′-dichlorodihydrofluorescein diacetate (CM-H2DCFDA). After the treatment with CM-H2DCFDA, the cells were washed twice with PBS and held at 37°C in PBS containing Ca2^+^, Mg2^+^, and H2DCFDA (10 μg/ml; Merck). After 30 min, the cells were washed again and analyzed using flow cytometry.

### Measurement of ROS

The intracellular ROS levels of the hepatocytes were quantified using an ROS Detection Assay Kit (Beyotime). Then, the hepatocytes were exposed to 10 μM DCFH-DA for 20 min at 37°C in a dark room. Subsequently, the hepatocytes were washed twice and photographed under a fluorescence microscope.

### Statistical analysis

All data are expressed as the mean ± standard deviation (SD). A one-way analysis of variance (ANOVA), complemented with the Tukey-Kramer multiple comparison test of equal sized samples, was performed to compare the data in the experimental groups with the data in the control group. All the statistical analyses were performed using a commercially available statistical software package (SPSS15.0, SPSS Inc, USA).
